# Correlations of visfatin with severity of acute myocardial infarction, cardiovascular risk factors and atrial fibrillation after percutaneous coronary intervention

**DOI:** 10.5937/jomb0-41963

**Published:** 2023-10-27

**Authors:** Huihui Zhang, Dingwei Lei, Miaolin Zhang, Sijia Tu, Chaofeng Shen, Fengxian Lin

**Affiliations:** 1 First People's Hospital of Linping District, Department of Cardiovascular Medicine, Hangzhou, China; 2 Affiliated Hospital of Chengdu University of Traditional Chinese Medicine, Department of Cardiovascular Medicine, Chengdu, China; 3 First People's Hospital of Linping District, Department of Orthopedic Surgery, Hangzhou, China; 4 The Third Affiliated Hospital of Wenzhou Medical University, Department of Cardiovascular Medicine, Wenzhou, China

**Keywords:** acute myocardial infarction, visfatin, cardiovascular risk factors, atrial fibrillation, akutni infarkt miokarda, visfatin, kardiovaskularni faktori rizika, atrijalna fibrilacija

## Abstract

**Background:**

To investigate the relationship between visfatin level in the peripheral blood of patients with acute myocardial infarction (AMI) patients and the severity of AMI, cardiovascular risk factors and atrial fibrillation after percutaneous coronary intervention (PCI).

**Methods:**

A total of 37 AMI patients diagnosed and treated in our hospital were selected as experimental group, and 35 patients with normal coronary angiography were enrolled as control group. The general pathological data and occurrence of atrial fibrillation after PCI of all the patients were recorded in detail, and the content of indexes related to the severity of AMI and visfatin was measured. Moreover, the correlations of visfatin with the severity of AMI, cardiovascular risk factors and atrial fibrillation after PCI were explored.

## Abstract (sr)

**Uvod:**

Cilj je bio da se istraži veza između nivoa visfatina u perifernoj krvi pacijenata sa akutnim infarktom miokarda (AMI) i težine AMI, kardiovaskularnih faktora rizika i atrijalne fibrilacije nakon perkutane koronarne intervencije (PCI).

**Metode:**

U eksperimentalnu grupu izabrano je ukupno 37 pacijenata sa AIM dijagnostikovanim i lečenim u našoj bolnici, a kao kontrolna grupa 35 pacijenata sa normalnom koronarografijom. Detaljno su evidentirani opšti patološki podaci i pojava atrijalne fibrilacije nakon PCI kod svih pacijenata i meren je sadržaj indeksa koji se odnose na težinu AMI i visfatina. Staviše, istražene su korelacije visfatina sa težinom AMI, kardiovaskularnim faktorima rizika i atrijalnom fibrilacijom nakon PCI.

**Rezultati:**

Razlike u nivoima N-terminalnog pro-B-tipa natriuretičkog peptida (NT-proBNP), srčanog troponina I (cTnI), mioglobina (MIO) i mase izoenzima kreatin kinaze MB (CK-MBm) u perifernoj krvi, krajnji sistolni prečnik leve komore (LVED), krajnji dijastolni volumen leve komore (LVEDV) i ejekciona frakcija leve komore (LVEF) bili su statistički značajni između eksperimentalne i kontrolne grupe. Staviše, eksperimentalna grupa je imala značajno viši nivo visfatina u perifernoj krvi od kontrolne grupe. Nivo visfatina u perifernoj krvi bio je u pozitivnoj korelaciji sa težinom AMI i kardiovaskularnim faktorima rizika, ali je bio negativno povezan sa atrijalnom fibrilacijom nakon PCI.

**Zaključak:**

Nivo visfatina u perifernoj krvi pacijenata sa AIM ima blisku korelaciju sa težinom AIM, kardiovaskularnim faktorima rizika i atrijalnom fibrilacijom posle PCI. Ova studija sugeriše da merenje nivoa visfatina u perifernoj krvi pacijenata sa AIM može pružiti korisne informacije o težini AMI, kardiovaskularnim faktorima rizika i verovatnoći atrijalne fibrilacije nakon PCI. Stoga, visfatin može poslužiti kao potencijalni biomarker za stratifikaciju rizika i personalizovani tretman pacijenata sa AIM.

## Introduction

Acute myocardial infarction (AMI) is a critical coronary heart disease that can lead to life-threatening complications such as arrhythmia, heart failure, and cardiogenic shock [1] [2] [3]. The disease is primarily caused by inadequate blood and oxygen supply to the myocardium due to coronary artery stenosis induced by factors in vivo and in vitro, which can result in myocardial necrosis in severe cases. However, with the continuous development of contemporary medical technologies and constant improvement in diagnostic and therapeutic means for AMI, the mortality rate of the disease has been reduced remarkably through multiple medico-surgical management measures, including new drugs and percutaneous coronary intervention (PCI). Research evidence indicates that atrial fibrillation often occurs in AMI patients after PCI, which is a primary factor influencing the effective rate of treatment and postoperative quality of life of such patients [4] [5]. However, currently, there are no definite peripheral blood indexes for evaluating the severity of AMI, the cardiovascular risk factors, and the incidence rate of atrial fibrillation after PCI.

Visfatin is a member of the adipokine family, a highly conserved protein with a molecular weight of 52 kD that mainly regulates metabolism, immunity, and inflammation [6]. Studies have shown that visfatin can promote the release of inflammatory factors, lead to vascular endothelial injury, induce the rupture of unstable atheromatous plaques, and participate in regulating the occurrence and development of coronary heart disease [7]. This research aims to analyze the relations of visfatin in the peripheral blood with the severity of AMI, the cardiovascular risk factors, and the incidence rate of atrial fibrillation after PCI. The objective is to provide theoretical bases for guiding the diagnosis and treatment of AMI patients and evaluating their prognosis in the clinic.

## Materials and methods

### Research subjects

AMI patients diagnosed and treated in our hospital were selected. All the enrolled patients were definitely diagnosed through coronary angiography, echocardiography and examination of markers for myocardial necrosis. AMI was diagnosed in accordance with the diagnostic criteria formulated by the American Heart Association, the American College of Cardiology and the European Society of Cardiology in 2012 [8]. Specifically, AMI is diagnosed when there is clinical evidence of myocardial necrosis in a clinical setting consistent with acute myocardial ischemia, and any one of the following criteria should be met: (1) The level of at least one cardiac biomarker rises or declines by over 99% of the upper limit of normal reference value, accompanied with at least one of the changes hereinafter: a) pathological Q waves in electrocardiogram, b) symptoms of myocardial ischemia, c) new regional wall motion abnormalities or lack of viable myocardium in imaging examination and d) new-onset left bundle branch block or apparent ST-T segment changes: (2) There are concomitant ischemic changes on the electrocardiogram and symptoms of myocardial ischemia or emerging cardiac death caused by left bundle branch block (3). There are coronary ischemic events associated with coronary artery bypass grafting: (4) There are PCI-related coronary ischemic events: (5) There is AMI-related stent thrombosis according to autopsy or coronary angiography. Experimental group consisted of 37 patients aged 52-79 years old, including 20 males and 17 females. Patients with other chronic wasting diseases, hyperthyroidism or hypothyroidism, malignant tumors or hematological diseases were excluded from this research. Besides, a total of 35 patients (50-77 years old) with normal coronary angiography in the same time period were enrolled as control group, including 18 males and 17 females. Signed written informed consents were obtained from all participants before the study. This study was approved by the Ethics Committee of First People's Hospital of Linping District.

### Collection of medical history and physical examination

The age, gender, past medical history and history of smoking and drinking of the enrolled patients were collected, and the previous administration of drugs and other basic situations were recorded. In addition, the blood pressure, body height and body weight of each patient was measured and recorded at admission, and the body mass index (BMI) was calculated.

### Laboratory examinations

Echocardiography (probe frequency: 3-11 MHz) was performed for all patients by the same experienced ultrasound physician using a color Doppler ultrasonic diagnostic apparatus. With the American Society of Echocardiography guidelines as the measurement standards, the left ventricular end-systolic diameter (LVEDs), left ventricular end-diastolic volume (LVEDV) and left ventricular ejection fraction (LVEF) were recorded.

All participants were examined by coronary angiography conducted by the same cardiac intervention physician using Judkins method, so as to independently evaluate the severity of coronary arterial lesions. The coronary arterial lesions were classified into single-vessel, double-vessel and multi-vessel coronary artery disease, and the left main disease alone was recorded as double-vessel coronary artery disease.

Fasting venous blood (5 mL) was gathered from every patient, let stand at 25°C for 30 min and centrifuged at 4°C and 3,000 r/min for 10 min. Then the supernatant was obtained to detect biochemical indexes (fasting blood glucose, serum albumin (ALB) and total cholesterol (TC)) and measure the content of AMI-related indexes (N-terminal pro-B-type natriuretic peptide (NT-proBNP), cardiac troponin I (cTnI) and creatine kinase isoenzyme MB mass (CK-MBm)) in each patient.

### Detection of visfatin level in peripheral blood

A total of 5 mL of fasting venous blood was collected from each patient, placed at room temperature for 30 min and centrifuged at 4°C and 3,000 r/min for 10 min. Subsequently, the plasma was collected to determine the content of visfatin in the peripheral blood via Human Visfatin Enzyme-linked immunosorbent assay (ELISA) Kit (Phoenix Pharmaceuticals, Burlingame, CA, USA) strictly according to the instructions. Finally, the absorbance was detected to calculate the content of visfatin.

### Follow-up

The patients were followed up during hospitalization or through telephone interview based on their medical records, and the disease progression and survival time of the patients were recorded. Next, the patients were divided into low concentration group (visfatin concentration 7.50 µg/L) and high concentration group (visfatin concentration >7.50 µg/L), and with the occurrence of major adverse cardiovascular events as the endpoint, the detailed records of follow-up and various examinations were preserved.

### Statistical analysis

The data in this research were presented as mean ± standard deviation and analyzed using Statistical Product and Service Solutions (SPSS) 22.0 software (IBM, Armonk, NY, USA). The measurement data were analyzed by using the t-test, and the categorical data were analyzed via χ^2^ test. Differences between two groups were adopted by using the independent-samples t-test, and one-way analysis of variance was utilized for comparison among multiple groups. The data were subjected to homogeneity test of variance, Bonferroni method was used for pairwise comparison of data showing equal variance, and the data with unequal variance were analyzed through Welch method. Besides, Dunnett's T3 test was performed for multiple comparisons. The follow-up and survival of the patients were assessed via Kaplan-Meier analysis, and the correlations among indexes were evaluated by means of Spearman correlation analysis. P<0.05 indicated the significant difference.

## Results

### General data of patients

The general data of the patients in control group and experimental group were recorded in detail. As shown in Table 1, there were no statistically significant differences in the age, gender and TC between two groups (P>0.05). However, the differences in the histories of smoking and drinking, hypertension, diabetes mellitus and creatinine (Cr) were statistically significant between two groups (P<0.05).

**Table 1:** General data of patients.

Group	Control group<br>(n=35)	Experimental<br>group (n=37)	P
Age (years old)	63.32±7.93	65.98±10.53	0.089
Gender (male/female)	(18/17)	(20/17)	
BMI (kg/m^2^)	22.97±3.08	23.11±2.86	0.073
Smoking (n (%))	11 (31.43%)	19 (51.35%)	0.006
Drinking (n (%))	12 (34.29%)	22 (59.50%)	0.021
Hypertension (n (%))	9 (25.72%)	24 (64.86%)	0.003
Diabetes mellitus (n (%))	10 (28.57%)	21 (56.76%)	0.004
ALB (g/L)	41.12±3.67	40.68±2.39	0.238
TC (mmol/L)	4.35±0.87	4.22±1.08	0.118
Cr (mol/L)	70.18±16.28	82.33±20.19	0.026

### Changes in levels of indexes related to severity of AMI and echocardiography parameters

The changes in the content of AMI-associated indexes in the peripheral blood and echocardiography parameters in fasting state were detected in control group and experimental group. The results indicated that the content of NT-proBNP, cTnI, MYO and CK-MBm in the peripheral blood, LVEDs, LVEDV and LVEF had statistically significant differences between experimental group and control group (*P*<0.05) (Table 2).

**Table 2:** Changes in levels of indexes related to severity of AMI and echocardiography parameters. Note: P<0.01 vs. control group.

Group	Control group<br>(n=35)	Experimental<br>group (n=37)	P
NT-proBNP<br>(ng/L)	139.33±39.27	839.33±119.28	0.002
CTnI (µg/L)	0.03±0.01	21.19±15.82	0.000
MYO (µg/L)	33.28±11.35	289.17±182.59	0.000
CK-MBm<br>(µg/L)	0.69±0.38	82.63±59.16	0.000
LVEDs (mm)	25.92±5.88	32.78±6.92	0.027
LVEDV (mL)	83.98±29.60	96.31±38.16	0.003
LVEF (%)	63.53±5.72	52.91±3.98	0.001

### Content of visfatin in peripheral blood

The content of visfatin in the fasting peripheral blood of patients in each group was measured using the ELISA kit, and it was manifested that the content of visfatin in the peripheral blood was significantly higher in experimental group than that in control group, with a significant difference (P<0.01) (Table 3). Moreover, the content of visfatin was raised markedly with the increase in the number of diseased coronary vessels in patients in experimental group.

**Table 3:** Content of visfatin in peripheral blood of patients.

Group	Visfatin (ug/L)	P
Control group	6.74±1.39	
Experimental group	8.83±1.93	0.009
Single-vessel coronary artery<br>disease	7.32±2.18	0.011
Double-vessel coronary artery<br>disease	8.06±1.93	0.010
Multi-vessel coronary artery<br>disease	9.21±2.39	0.007

### Correlation analysis of visfatin with severity of AMI

**Figure 1:**
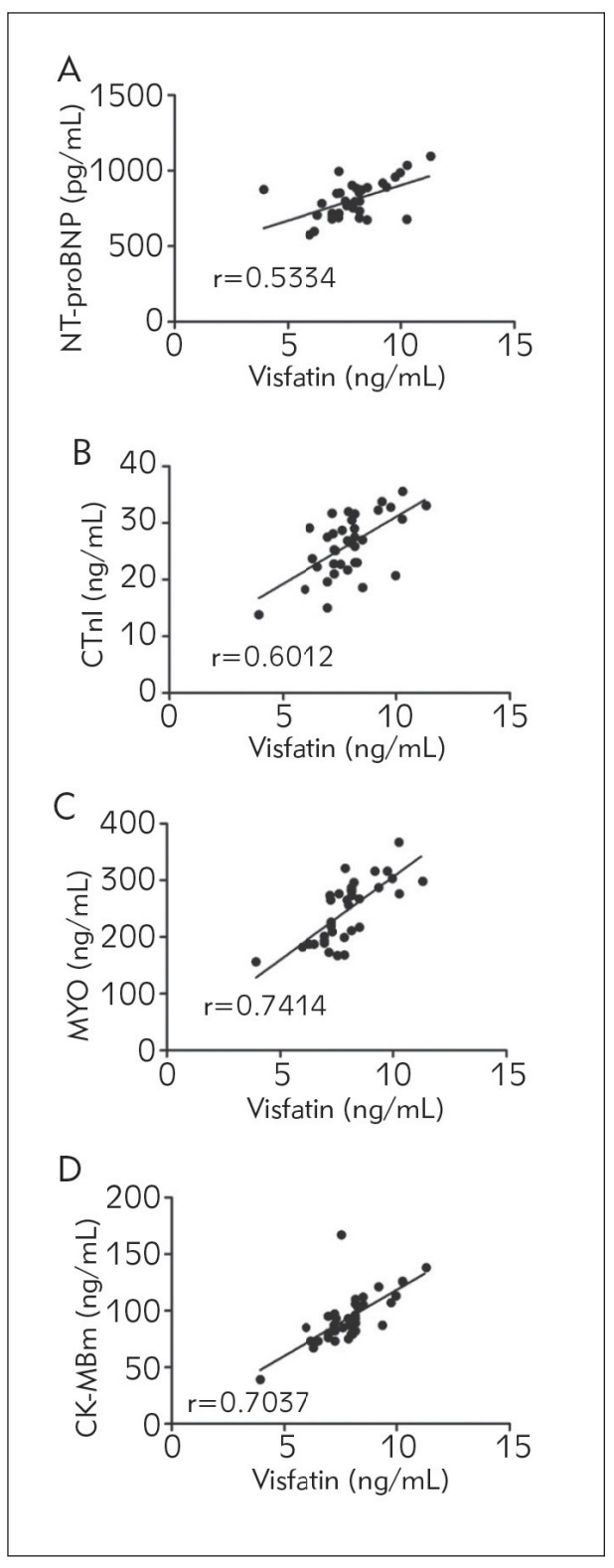
Correlation analysis of visfatin with severity of AMI. (A) Correlation between visfatin and NT-proBNP. (B) Correlation between visfatin and CTnI. (C) Correlation between visfatin and MYO. (D) Correlation between visfatin and CK-MBm. The content of visfatin in the peripheral blood was positively correlated with NT-proBNP, CTnI, MYO and CK-MBm in AMI patients (P<0.01).

The associations of visfatin in the peripheral blood with NT-proBNP, CTnI, MYO and CK-MBm in patients were recorded, so as to evaluate the correlation between visfatin and the severity of AMI. The level of visfatin in the peripheral blood of AMI patients was positively correlated with NT-proBNP (r=0.5334, P<0.01), CTnI (r=0.6012, P<0.01), MYO (r=0.7414, P<0.01) and CK-MBm (r=0.7037, P<0.01), that is, the content of visfatin in the peripheral blood had a positive correlation with the severity of AMI (Figure 1).

### Correlations of visfatin with cardiovascular risk factors

**Figure 2:**
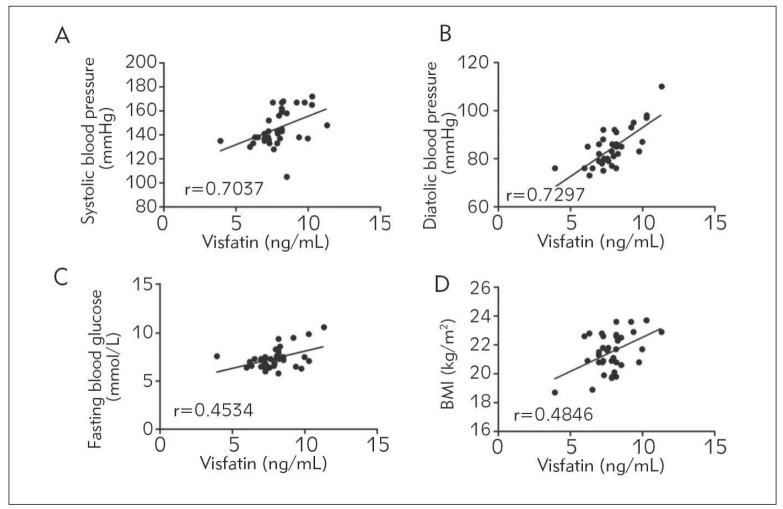
Correlation analysis of visfatin with cardiovascular risk factors. (A) Correlation between visfatin and systolic blood pressure. (B) Correlation between visfatin and diastolic blood pressure. (C) Correlation between visfatin and fasting blood glucose. (D) Correlation between visfatin and BMI. The content of visfatin in the peripheral blood was positively correlated with the systolic blood pressure, diastolic blood pressure, fasting blood glucose and BMI in AMI patients (P<0.01).

The relationships between visfatin in the peripheral blood and the blood pressure in patients, blood glucose and Cr were recorded to analyze the correlations of visfatin with the cardiovascular risk factors. The results manifested that visfatin in the peripheral blood exhibited positive correlations with systolic blood pressure (r=0.7037, P<0.01), diastolic blood pressure (r=0.7297, P<0.01), fasting blood glucose (r=0.4534, P<0.01) and BMI (r=0.4846, P<0.01) in patients. In other words, the content of visfatin in the peripheral blood was positively related to the cardiovascular risk factors (Figure 2).

### Correlation between visfatin and atrial fibrillation after PCI

**Figure 3:**
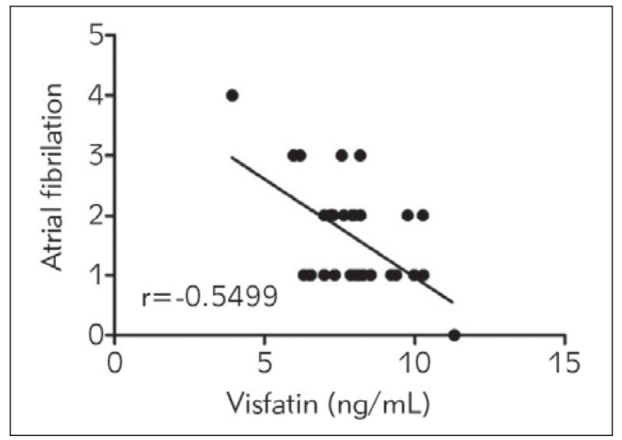
Correlation between visfatin and atrial fibrillation after PCI. There was a negative correlation between visfatin in the peripheral blood and the incidence rate of atrial fibrillation after PCI in patients (P<0.01).

The correlation between visfatin in the peripheral blood of patients and the frequency of atrial fibrillation after PCI was recorded. It was revealed that there was a negative correlation between visfatin in the peripheral blood and the incidence rate of atrial fibrillation after PCI in patients (r=-0.5499, P<0.01) (Figure 3).

## Discussion

AMI is a critical disease of coronary atherosclerosis, with its major pathogenic factor being secondary thrombosis caused by the rupture and erosion of unstable plaques in the coronary artery. This results in coronary occlusion and myocardial ischemia and necrosis [9]. Visfatin, a type of adipocytokine, has multiple biological effects and is closely associated with the occurrence and development of hypertension, heart failure, and coronary heart disease according to a large amount of research evidence in recent years. Visfatin is highly expressed in ruptured coronary atherosclerotic plaques and macrophages [10] [11].

A study by Hofer et al. [12] found that the content of visfatin is positively correlated with C-reactive protein and triglycerides in patients with coronary heart disease. Furthermore, it interacts with C-reactive protein. Echocardiogram and NT-proBNP are the most commonly used indexes to evaluate the changes in cardiac systolic function, which are highly related to the severity of myocardial infarction [13].

In this research, the levels of visfatin, NT-proBNP, CTnI, MYO, and CK-MBm in the peripheral blood of AMI patients were measured, and their correlations were analyzed. The results revealed that the levels of visfatin, NT-proBNP, CTnI, MYO, and CK-MBm in the peripheral blood of AMI patients were significantly higher than those of normal people. Moreover, the content of visfatin increased as the number of diseased coronary vessels in AMI patients increased. Additionally, the level of visfatin in the peripheral blood of patients with AMI was positively correlated with the levels of NT-proBNP, CTnI, MYO, and CK-MBm. These results strongly suggest that there is an intimate correlation between visfatin and the severity of AMI. Massive amounts of visfatin are released into the blood circulation in AMI patients, which significantly increases the release of inflammatory factors such as interleukin-6 and interleukin-1. This enhances the DNA binding activity of nuclear factor-B and promotes the expression of matrix metalloproteinases, thereby leading to endothelial dysfunction and increasing the instability of atheromatous plaques. It can be seen that the high levels of visfatin in AMI patients further facilitate the development of myocardial infarction by affecting the vascular endothelial function [14] [15].

Yamamoto et al. [16] discovered through research that smoking, hypertension, diabetes mellitus and BMI are traditional risk factors for coronary heart disease, and visfatin combined with such risk factors can effectively predict the occurrence and development of AMI. The relations of cardiovascular risk factors with visfatin were analyzed in this research, and it was indicated that the blood pressure, blood glucose and BMI had positive correlations with visfatin in the peripheral blood. Furthermore, survival analysis was conducted for AMI patients, and the results showed that AMI patients in high concentration group had a prominently shorter survival time than those in low concentration group. A large amount of extracellular visfatin can release numerous inflammatory factors, which causes the rupture of unstable atheromatous plaques, increases the incidence rate of adverse cardiovascular events in AMI patients and distinctly shortens the survival time of the patients [17]. Atrial fibrillation is currently one of the most common types of persistent arrhythmia in clinic, and PCI-induced atrial fibrillation is closely associated with AMI, whose risk can be reduced remarkably by administration with anticoagulants and antiplatelet agents [18] [19]. It was found in this research that the content of visfatin in the peripheral blood of AMI patients had a negative correlation with the incidence rate of atrial fibrillation after PCI, implying that the occurrence of atrial fibrillation after PCI can be predicted by detecting the content of visfatin in patients. According to the study of Platek et al. [20], the content of plasma visfatin varies among patients with different types of atrial fibrillation, and the progression of atrial fibrillation can clearly elevate the level of visfatin. Despite the strengths of our study, there are several limitations that should be taken into consideration. Firstly, this study was conducted in a single center and had a relatively small sample size, which may limit the generalizability of the results. Secondly, our study only measured visfatin levels at a single time point and did not follow up with patients over a longer period of time to assess changes in visfatin levels and their impact on outcomes. Thirdly, we did not assess other potential confounding factors, such as medications or comorbidities that may influence visfatin levels and cardiovascular outcomes. Fourthly, our study did not include a control group, which could have provided further insight into the associations between visfatin levels and outcomes. Finally, although our study found an association between visfatin levels and cardiovascular outcomes, we cannot infer causality from our cross-sectional study design. Further studies with larger sample sizes, longer follow-up periods, and more comprehensive assessments of potential confounding factors are needed to confirm and expand upon our findings.

## Conclusions

In conclusion, our study highlights the significant association between visfatin levels and the severity of AMI, as well as its correlation with various cardiovascular risk factors and the incidence of AF after PCI. Our findings suggest that visfatin may serve as a potential biomarker for risk stratification and prognosis assessment in AMI patients undergoing PCI. Furthermore, the results of this study provide a basis for further investigations into the underlying mechanisms linking visfatin to the pathogenesis of AMI and AF, which may lead to the development of novel therapeutic strategies for these conditions. Overall, our study contributes to the growing body of literature on the role of visfatin in cardiovascular disease and underscores the importance of considering visfatin as a potential therapeutic target.

## Dodatak

### Conflict of interest statement

All the authors declare that they have no conflictof interest in this work.
